# Porcine Epidemic Diarrhea Virus Is Inhibited by GS-441524 During an In Vitro Infection

**DOI:** 10.3390/microorganisms13051089

**Published:** 2025-05-08

**Authors:** Shijuan Dong, Rujing Sun, Bingqing Chen, Fusheng Si, Chunhua Li, Daojing Zhang, Ruisong Yu, Huili Liu

**Affiliations:** 1 Institute of Animal Science and Veterinary Medicine, Shanghai Key Laboratory of Agricultural Genetics and Breeding, Shanghai Engineering Research Center of Breeding Pig, Shanghai Academy of Agricultural Sciences (SAAS), Shanghai 201106, China; dsjuan@saas.sh.cn (S.D.); chenbingqing@saas.sh.cn (B.C.); mr.fusheng@163.com (F.S.); lichunhua@saas.sh.cn (C.L.); 2 State Key Laboratory of Bioreactor Engineering, East China University of Science and Technology (ECUST), Shanghai 200237, China; sun2796872450@163.com (R.S.); djz@ecust.edu.cn (D.Z.)

**Keywords:** porcine epidemic diarrhea virus (PEDV), GS-441524, antiviral, virus replication

## Abstract

Porcine epidemic diarrhea virus (PEDV), the etiology of porcine epidemic diarrhea (PED), continues to impose severe economic burdens on pig farms in China. The continuous emergence of new variant strains makes it difficult for vaccinated sows to provide protective immunity to piglets. Hence, there is an urgent need for efficacious therapeutic drugs in clinical practice. In the present study, the antiviral activity of GS-441524, a nucleoside analogue, against PEDV was evaluated. It can efficiently inhibit the proliferation of trypsin-dependent and trypsin-independent PEDVs in a dose-dependent manner, exhibiting greater efficacy against the trypsin-independent strain. GS-441524 can inhibit trypsin-independent PEDV proliferation in Vero cells with EC_50_ and CC_50_ values of 2.6 μM and 104.4 μM, respectively. As expected, GS-441524 exerts its inhibitory effect during the replication phase of the four stages of the PEDV proliferation cycle. Even at a high viral infection dose of MOI 0.5 or added 6 h post-viral infection, 20 μM GS-441524 can still effectively inhibit PEDV proliferation. These findings emphasize the potent antiviral activity of GS-441524 against PEDV, and its therapeutic efficacy on PEDV-infected piglets warrants further investigation.

## 1. Introduction

Porcine epidemic diarrhea virus (PEDV), a porcine enteropathogenic coronavirus belonging to the *Alphacoronavirus* genus in the *Coronaviridae* family, is the causative agent of porcine epidemic diarrhea (PED), causing acute diarrhea, vomiting, and dehydration in pigs, and leading to a high mortality in newborn piglets [1]. PEDV was initially isolated in Belgium in 1978 [2]. Since 2010, new highly virulent variants of PEDV have led to the pandemic of PED in China and gradually spread to regions across Asia, Europe, America, and beyond, inflicting substantial economic losses on pig farms worldwide [3,4,5]. In recent years, PED has continued to be highly prevalent and has been ranked among the most critical diseases in Chinese pig farms [6]. Currently, the prevention of PED mainly relies on vaccines, with antiviral drugs as effective supplements.

Among numerous anti-coronavirus drugs from various sources, nucleoside analogues, such as GS-441524, remdesivir, and molnupiravir, have shown broad-spectrum antiviral activity [7,8,9,10]. For example, GS-441524, the nucleoside precursor of remdesivir, has been proven highly efficacious against feline infectious peritonitis coronavirus (FIPV) and is well tolerated in cats [11]. GS-441524 also showed a similar efficacy to remdesivir in inhibiting the replication of SARS-CoV-2 [12]. Compared with remdesivir, GS-441524 has a number of pharmacological advantages, including needing fewer steps to be synthesized in vitro and be activated in vivo, less toxicity, which allows for higher concentrations of aerosol and oral administration, and better oral bioavailability [13,14]. Therefore, GS-441524 exerts more potent clinical value in the treatment of infectious diseases caused by coronaviruses. Recently, the anti-PEDV activities of GS-441524 and remdesivir were also tested. Xie et al. showed that GS-441524 exhibited stronger anti-PEDV activity and lower cytotoxicity than remdesivir in Vero cells and showed a good safety profile in cells and mice [15]. Although it is evident that GS-441524 and remdesivir exert their anti-PEDV capability by directly inhibiting the viral RNA-dependent RNA polymerase (RdRp) activity, there are still several issues worth clarifying. For example, the antiviral activity of GS-441524 against trypsin-independent PEDV is still unknown. Is there a significant difference in the antiviral activity of GS-441524 against trypsin-dependent and trypsin-independent PEDVs? The answer to this question requires further investigation into the impact of GS-441524 on PEDV proliferation. In this study, we utilized trypsin-dependent and trypsin-independent strains to further study the inhibitory activity of GS-441524 against different PEDV strains and its impact on the viral cycle. We found that GS-441524 significantly inhibited the replication of trypsin-dependent and trypsin-independent PEDV. Further experiments showed that GS-441524 affected replication stages of the viral life cycle, including viral genome amplification, viral protein expression, and virion production. These results indicate that GS-441524 can serve as a potential therapeutic alternative for PED control.

## 2. Materials and Methods

### 2.1. Cells, Virus, and Compound

Vero CCL-81 cells (African green monkey kidney cells, purchased from ATCC) were cultured in Dulbecco’s Modified Eagle Medium (DMEM) (Hyclone, Logan, UT, USA), supplemented with 10% fetal bovine serum (FBS) (Gibco BRL, Gaithersburg, MD, USA), 100 U/mL of penicillin, and 100 μg/mL of streptomycin, at 37 °C in a 5% CO_2_-enriched atmosphere. The trypsin-independent attenuated PEDV DR13^att^ (JQ023162; isolated from a commercial vaccine of Green Cross, Yongin, Republic of Korea), rPEDV-∆ORF3-GFP (recombinant PEDV DR13^att^ with ORF3 gene replaced by GFP gene) [16], and trypsin-dependent rPEDV-S (recombinant PEDV DR13^att^ with S gene replaced by S gene from a trypsin-dependent strain (KF650375) were propagated in Vero cells. Virus titers were determined using the Reed-Muench method [17] and expressed as tissue culture infective dose 50% (TCID_50_). GS-441524 (Yuanye, Shanghai, China) was dissolved in 100% dimethylsulfoxide (DMSO) to form a 10 mM stock solution and diluted with DMEM to the specified concentrations before use.

### 2.2. Cytotoxicity Determination

The cytotoxicity of GS441524 was determined in Vero cells using the MTT assay. The cells were seeded at a density of 2 × 10^4^ cells/well into 96-well plates. When the cells reached 90% confluence within about 24 h, the supernatant was discarded, and the cells were washed three times with PBS. Subsequently, two-fold serial diluted GS-441524, starting from an initial concentration of 400 μM, was added to the cells and incubated for 3 d, with six replicates for each concentration. Cell control and vehicle (DMSO) control were set up. The cell viability was detected using the MTT assay. In brief, 20 μL MTT solution (5 mg/mL) was added to each well and incubated at 37 °C for 4 h. Then, the reaction solution was removed, and 150 μL DMSO was added to each well, followed by slow shaking of the plate at room temperature for 10 min. The optical density (OD) of each well was measured at a wavelength of 490 nm. The relative viability of the cells was calculated according to the formula: cell survival rate (%) = OD (sample)/OD (control) × 100%. The half cytotoxic concentration (CC_50_) of GS-441524 was calculated.

### 2.3. Determination of the Half Maximal Effective Concentration (EC_50_) of GS-441524

The half maximal effective concentration (EC_50_) of GS-441524 against trypsin-independent PEDV DR13^att^ was determined with Vero cells cultured in 96-well plates. Briefly, Vero cells with 90% confluency were infected with DR13^att^ at a multiplicity of infection (MOI) of 0.01 and cultured for 3 d in the presence of two-fold serial dilutions of GS-441524. Cell control (without virus and compound), virus control (without compound and vehicle), and vehicle (DMSO) control were set up. The cell viability was determined using the MTT assay. The relative inhibitory rate of GS-441524 on PEDV was calculated according to the following formula: inhibitory rate (%) = [OD (sample) − OD (virus control)]/[OD (cell control) − OD (virus control)] × 100%, and the EC_50_ of GS-441524 was calculated.

### 2.4. Antiviral Effect of GS-441524 Against Trypsin-Independent PEDV

To analyze the inhibitory effect of GS-441524 on PEDV, Vero cells inoculated with PEDV DR13^att^ were treated with different concentrations of GS-441524. The expression of PEDV (M and N) proteins was detected using IFA or Western Blot, and the viral titers in the supernatant were determined. The detailed protocols were as follows.

For IFA analysis, Vero cells were seeded into 48-well plates at 4 × 10^4^ cells/well and cultured for about 24 h. Then, the cells were inoculated with PEDV DR13^att^ at MOI 0.1. The inoculum was removed after 2 h incubation, and fresh maintenance medium containing 0, 1.25, 2.5, 5, 10, and 20 μM GS-441524 was added. The cells were fixed at 24 h post inoculation (hpi) and underwent immunofluorescent staining to analyze the expression of the M protein.

For Western Blot and virus titration analysis, Vero cells were seeded into 6-well plates at 4 × 10^5^ cells/well. After about 24 h, the cells were inoculated with PEDV DR13^att^ at an MOI of 0.1. The inoculum was removed after 2 h incubation, and fresh maintenance medium containing 0, 1.25, 2.5, 5, 10, and 20 μM GS-441524 was added. The viral titers (TCID_50_) and the mRNA levels of M protein in the supernatant were determined at 12, 18, 24, and 30 hpi. The cells collected at 30 hpi were lysed and analyzed for N protein expression by Western Blot.

### 2.5. Comparison of the Antiviral Activity of GS-441524 Against Trypsin-Dependent and -Independent PEDVs 

Unlike cell-adapted strains that do not require trypsin, most clinical strains of PEDV require additional trypsin for proliferation. To further explore the anti-PEDV activity of GS-441524, Vero cells infected with DR13^att^ (trypsin-independent) and rPEDV-S (trypsin-dependent, by substituting the S gene of DR13^att^ with that of a trypsin-dependent strain) were treated with different concentrations of GS-441524. Cytopathic effects were observed, and the supernatant was collected at 24 and 48 hpi for viral titration (TCID_50_).

### 2.6. Effects of GS-441524 on the Life Cycle Phases of Trypsin-Independent PEDV

To assess the impact of GS-441524 on the life cycle of PEDV, Vero cells (1 × 10^5^ cells/well) were seeded into 24-well plates and cultured until 90% confluence. Then, the Vero cells were treated with GS-441524 according to the following protocols. For attachment, the cells were incubated with rPEDV-∆ORF3-GFP (MOI 0.01) and 20 μM GS-441524 at 4 °C for 2 h. Then, the inoculum was removed, and the cells were cultured with fresh DMEM for another 48 h at 37 °C. For entry, the cells were infected with rPEDV-∆ORF3-GFP at MOI 0.01 at 4 °C for 2 h. Then, the inoculum was discarded, and the cells were treated with 20 μM GS-441524 at 37 °C for 2 h. After removing GS-441524, the cells were further cultured with fresh DMEM at 37 °C for 46 h. For replication, the cells were incubated with rPEDV-∆ORF3-GFP at MOI 0.01 at 4 °C for 2 h. After discarding the inoculum, the cells were cultured in fresh DMEM for another 6 h at 37 °C. Then, GS-441524 was added to the culture up to 20 μM and incubated for another 42 h. For release, the cells were infected with rPEDV-∆ORF3-GFP at MOI 0.01 at 4 °C for 2 h, and then cultured at 37 °C for 44 h. Then, the supernatant was discarded, and the cells were cultured in fresh DMEM with 20 μM GS-441524 for another 4 h. To determine the capability of the drug to inactivate PEDV, rPEDV-∆ORF3-GFP was preincubated with GS-441524 at 37 °C for 1 h. Following a 2 h incubation at 4 °C with the cells, the inocula were removed, and the cells were further cultured in fresh DMEM at 37 °C for 48 h. Lastly, the viral titers and the mRNA levels of M protein in the supernatant were determined for samples collected after each treatment. Images of cells were captured using an EVOS fluorescence microscope (M7000, Thermo Fisher Scientific, Waltham, MA, USA).

### 2.7. Immunofluorescence Assay (IFA)

For immunofluorescence staining, PEDV-infected cells were washed twice with PBS, fixed with 4% paraformaldehyde for 15 min, and then permeabilized with 0.1% Triton X-100 in PBS at room temperature for 15 min. After being blocked with 5% bovine serum albumin (BSA), the cells were incubated with anti-PEDV M polyclonal antibody (1:100) for 1 h, then rinsed three times with PBS and incubated with Alexa Fluor 488-conjugated goat anti-rabbit antibody (1:200, Beyotime, Shanghai, China) for 1 h. The nuclei were visualized by DAPI nuclear staining. Images of the immunofluorescent cells were captured using an EVOS fluorescence microscope at a magnification of 200×.

### 2.8. Western Blot

The cells were lysed in RIPA lysis buffer supplemented with a protease inhibitor cocktail (TransGen Biotech, Beijing, China) and subjected to sodium dodecyl sulphate–polyacrylamide gel electrophoresis (SDS–PAGE). The proteins separated by SDS–PAGE in the gel were then transferred onto a polyvinylidene fluoride (PVDF) membrane. Next, the membrane was blocked with 5% non-fat milk in Tris-buffered saline-Tween (TBST) and incubated with either anti-PEDV N monoclonal antibody (1:3000, Shanghai Ango Biotechnology Ltd., Shanghai, China) or anti-GAPDH monoclonal antibody (1:2000, Sangon Biotech, Shanghai, China) at room temperature for 1 h. Finally, the membrane was incubated with horseradish peroxidase (HRP) conjugated goat anti-rabbit IgG (1:20,000, Sangon Biotech, Shanghai, China). The proteins were detected using the Amersham ECL Western Blotting Analysis System (GE Healthcare, Chicago, IL, USA).

### 2.9. RNA Extraction and Real-Time RT-PCR

Total RNA was extracted from cells using the TIANamp viral RNA kit (TIANGEN, Beijing, China). Subsequently, the extracted RNA was subjected to reverse transcription with reverse transcription reagent (Promega, Madison, WI, USA). The standards for SYBR Green real-time RT-PCR were produced by amplifying a 486 bp membrane (M) gene fragment and then cloning it into the pJET1.2/blunt vector (Thermo Fisher Scientific, Waltham, MA, USA) to construct a recombinant plasmid. The primers for standards (sense: 5′-TATTCCCGTTGATGAGGT-3′; antisense: 5′-GCAACCTTATAGCCCTCT-3′) and for qPCR (sense: 5′-TCTTGTGTTGGCACTGTCAC-3′; antisense: 5′-TGCAAGCCATAAGGATGCTG-3′) were synthesized by Sangon Company (Shanghai, China). Real-time RT-PCR was carried out using ABI 7500-fast Real-Time PCR systems (ABI, Foster City, CA, USA). Each 20 μL qPCR reaction system contained 2 μL reverse transcription sample, 10 μL TliRNaseH Plus (2×), 0.4 μL forward and reverse primers (10 μM), 0.4 μL ROX Reference Dye II (50×), and 6.8 μL sterile purified water. Amplification conditions were 95 °C for 30 s, followed by 40 cycles of 95 °C for 5 s and 60 °C for 34 s. All samples and standards were carried out in triplicate. The PEDV standards (recombinant plasmid) were diluted serially tenfold (10^1^ to 10^8^ copies/μL) to perform qPCR and establish a standard curve. The concentration of each sample was calculated by plotting the Ct value against the standard curve.

### 2.10. Statistical Analysis

Statistical analyses (one-way ANOVA) were performed using GraphPad Prism 8.0 software. All experiments were performed in three independent experiments, and data were expressed as mean values ± standard error of the mean (SEM). The statistical significances were defined as *p* < 0.05 (*), and the higher significance was denoted by *p* < 0.01 (**), *p* < 0.001 (***), and *p* < 0.0001 (****). Data relating to viral RNA copies and virus titers were converted to log10 to maintain a normal distribution.

## 3. Results

### 3.1. The Cytotoxicity of GS-441524 and Its Antiviral Activity Against Trypsin-Independent PEDV

**Figure 1:**
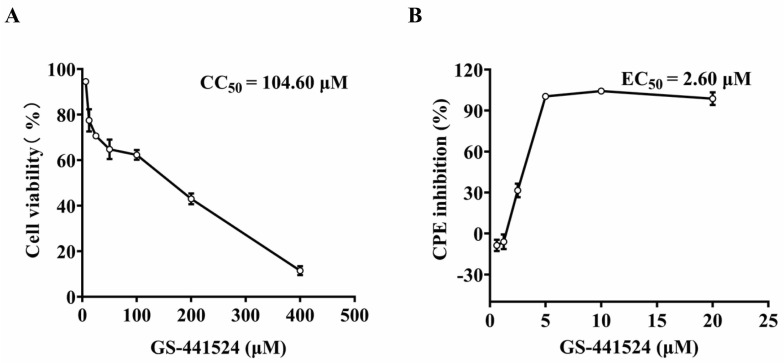
Cytotoxicity of GS-441524 and its antiviral activity against trypsin-independent PEDV. (**A**) Monolayer Vero cells were cultured for 3 d in DMEM containing two-fold serial dilutions of 400 μM GS-441524. Cell viability was evaluated using the MTT assay, and the CC_50_ value of GS-441524 was calculated. (**B**) Monolayer Vero cells were infected with PEDV DR13^att^ in the presence of two-fold serial diluted GS-441524 for 3 d. The cell viability was determined using the MTT assay, and the EC_50_ value of GS-441524 was calculated. Data are presented as the mean ± SEM of three independent experiments.

To determine the cytotoxicity of GS-441524 on the Vero cells, CC_50_ was determined by MTT assay. The CC_50_ value of GS-441524 was 104.4 μM (Figure 1A). To determine the antiviral activity of GS-441524, serially diluted drugs were added to the culture at 2 hpi with PEDV DR13^att^. The cytopathic effect (CPE) was obvious in the vehicle-treated Vero cells at 3 dpi, while the cells treated with 10 μM GS-441524 remained morphologically unchanged. In addition, GS-441524 had a dose-dependent effect on the alleviation of the CPE induced by the PEDV infection. The EC_50_ value of GS-441524 was determined as 2.6 μM (Figure 1B), leading to an excellent antiviral selectivity index value (SI) of 40.15 in Vero cells, suggesting that GS-441524 has potential anti-PEDV activities.

### 3.2. GS-441524 Inhibited Trypsin-Independent PEDV Proliferation in Vero Cells in a Dose-Dependent Manner

**Figure 2:**
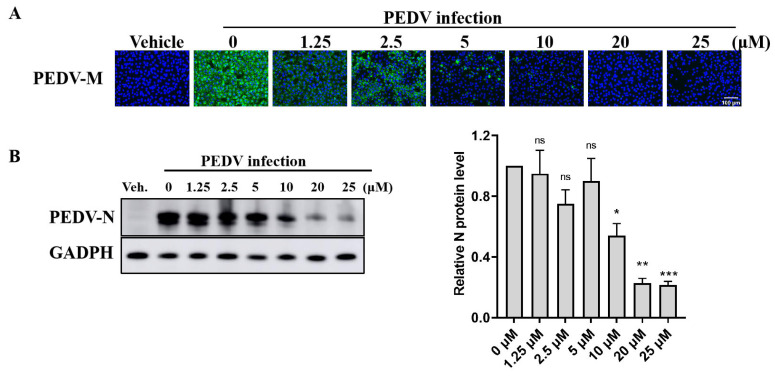
GS-441524 inhibits trypsin-independent PEDV proliferation in Vero cells in a dose-dependent manner. Vero cells were infected with PEDV DR13^att^ and treated with 0, 1.25, 2.5, 5, 10, and 20 μM GS-441524. (**A**) Vero cells were fixed and subjected to immunofluorescent staining, using anti-PEDV M polyclonal antibody at 24 hpi. The cell nucleus was stained with DAPI. Images were representative of results obtained from three independent experiments. Scale bars = 100 μm. (**B**) The cells were collected at 30 hpi and lysed for Western Blot. The quantification of Western Blot was performed using the GAPDH/PEDV-N ratio. The experiment was performed three times independently. Differences were considered significant at (*) *p* < 0.05, (**) *p* < 0.01, (***) *p* < 0.001. ns indicates non-significance.

**Figure 3:**
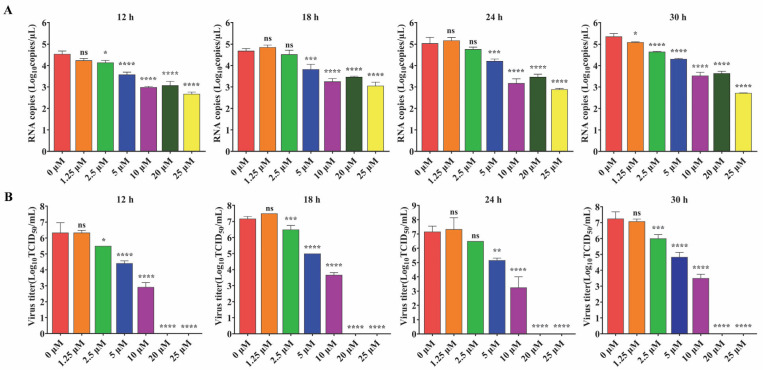
The effect of GS-441524 treatment on mRNA levels of PEDV M protein and PEDV titers at different time points. Vero cells were infected with trypsin-independent PEDV DR13^att^ and treated with 0, 1.25, 2.5, 5, 10, 20, and 25 μM GS-441524. Supernatants were collected at 12, 18, 24, and 30 hpi for the determination of mRNA levels of M protein (**A**) and viral titer (TCID_50_) (**B**). Data are presented as the mean ± SEM of three independent experiments. Differences were considered significant at (*) *p* < 0.05, (**) *p* < 0.01, (***) *p* < 0.001, (****) *p* < 0.0001. ns indicates non-significance.

To further explore the antiviral effect of GS-441524 on PEDV, the effects of different concentrations of the drug on the expression of PEDV structural proteins and viral titers were determined. The fluorescence intensity of M protein gradually decreased with the increase of GS-441524 concentration, and almost no fluorescence was observed when the drug concentration reached 20 μM, indicating that GS-44154 had a dose-dependent inhibitory effect on PEDV (Figure 2A). Similarly, as evidenced by Western Blot, the expression of N protein gradually declined with the increase of GS-441524 concentration (Figure 2B). The result of RNA levels of PEDV M protein at different time points under different drug concentrations showed that GS-441524 also significantly reduced RNA levels of M protein in a dose-dependent manner (Figure 3A). Consistent with the expression level of M and N protein, the viral titers were also significantly reduced by GS-441524 in a dose-dependent manner at distinct time points. Specifically, 5 μM GS-441524 could significantly inhibit the titers of PEDV at different time points when compared with the control group. Moreover, the viral titers could not be detected in the supernatant of the treatment group with 20 μM GS-441524 (Figure 3B).

### 3.3. Comparison of the Antiviral Activity of GS-441524 Against Trypsin-Dependent and -Independent PEDVs

**Figure 4:**
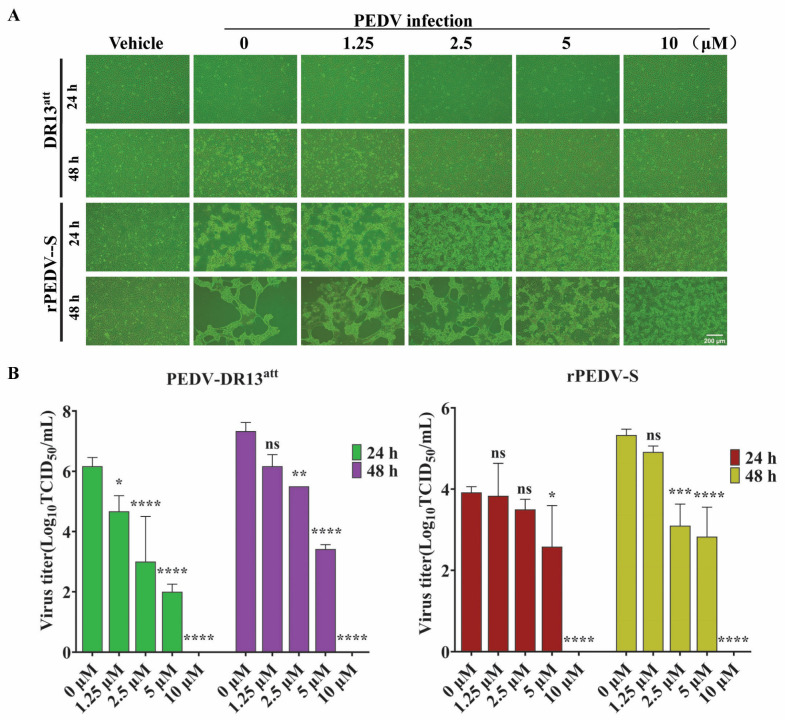
Comparison of the antiviral activity of GS-441524 against trypsin-dependent and -independent PEDVs. Vero cells were infected with DR13^att^ (trypsin-independent) and rPEDV-S (trypsin-dependent), and then GS-441524 was added at 2 hpi to final concentrations of 0, 1.25, 2.5, 5, and 10 μM, respectively. At 24 hpi and 48 hpi, cytopathy was observed (**A**), and the virus titers in the supernatant were determined (**B**). Data are presented as the mean ± SEM of three independent experiments. Differences were considered significant at (*) *p* < 0.05, (**) *p* < 0.01, (***) *p* < 0.001, (****) *p* < 0.0001. ns indicates non-significance.

To compare the inhibitory effect of GS-441524 on trypsin-dependent and -independent PEDVs, Vero cells were infected with DR13^att^ (trypsin-independent) or rPEDV-S (trypsin-dependent) strains in the presence of different concentrations of GS-441524. Compared with the control group, GS-441524 significantly inhibited the infections of both PEDV strains in a dose-dependent manner (Figure 4). The results of the cytopathy (Figure 4A) and viral titers (Figure 4B) at 24 hpi and 48 hpi showed that GS-441524 exhibited more potent antiviral activity against the trypsin-independent PEDV strain (DR13^att^) than against the trypsin-dependent rPEDV-S strain, especially at 24 hpi.

### 3.4. The Effect of GS-441524 on the Life Cycle of the Trypsin-Independent PEDV Strain

**Figure 5:**
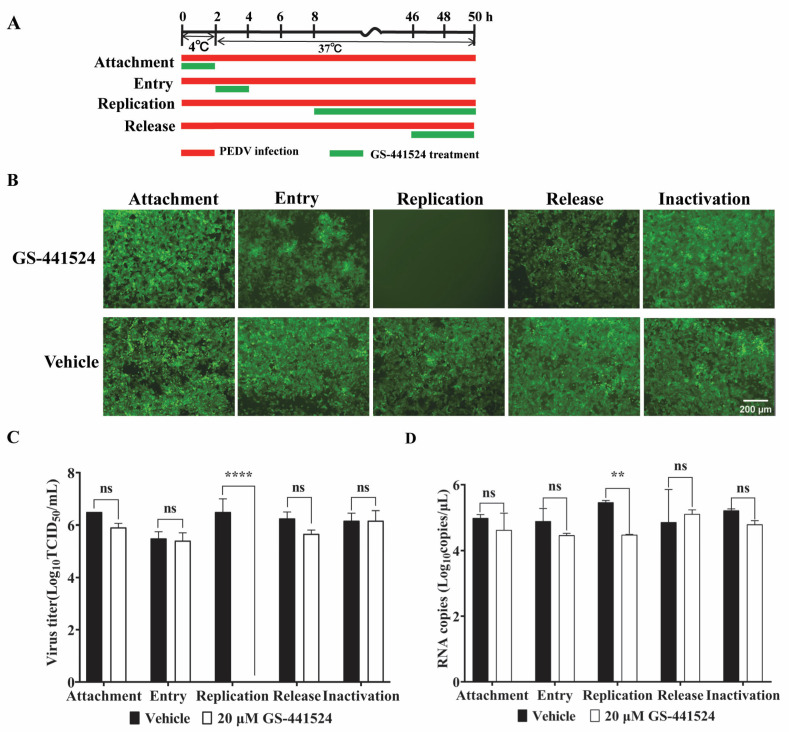
The effect of GS-441524 on the trypsin-independent PEDV life cycle. (**A**) Schematic diagram of GS-441524 treatment in different phases of PEDV proliferation. Red bars represent PEDV infection, and green bars represent GS-441524 treatment. (**B**–**D**) Vero cells were infected with rPEDV-∆ORF3-GFP and treated with 20 μM GS-441524 at indicated time points, which represented the stages of viral attachment, entry, replication, release, and inactivation, respectively. At 50 hpi, GFP expression of rPEDV-∆ORF3-GFP was observed. Scale bars = 200 μm. The cell culture supernatants were collected for the determination of viral titer (**C**) and mRNA level of M protein (**D**). Data are presented as the mean ± SEM of three independent experiments. Differences were considered significant at (**) *p* < 0.01, (****) *p* < 0.0001. ns indicates non-significance.

To evaluate the impacts of GS-441524 on the four phases of the PEDV life cycle, GS-441524 was added to Vero cell culture at different time points post rPEDV-GFP infection, as depicted in Figure 5A. The supernatant was then collected at 50 hpi. As expected, GS-441524 inhibited the replication phase of the PEDV life cycle, as evidenced by the absence of GFP fluorescence in the drug treatment group (Figure 5B). However, when administered during the attachment, entry, or release phases, there was no significant difference in the GFP signal in the cells compared with that in the vehicle group. In addition, GS-441524 showed no inactivation activity against PEDV. The results of viral titer determination and mRNA level assay also showed that GS-441524 significantly impeded the virus replication, while it had no effect on the attachment, entry, and release processes of PEDV (Figure 5C,D). Collectively, these results indicate that GS-441524 inhibits the proliferation of PEDV by blocking viral replication.

**Figure 6:**
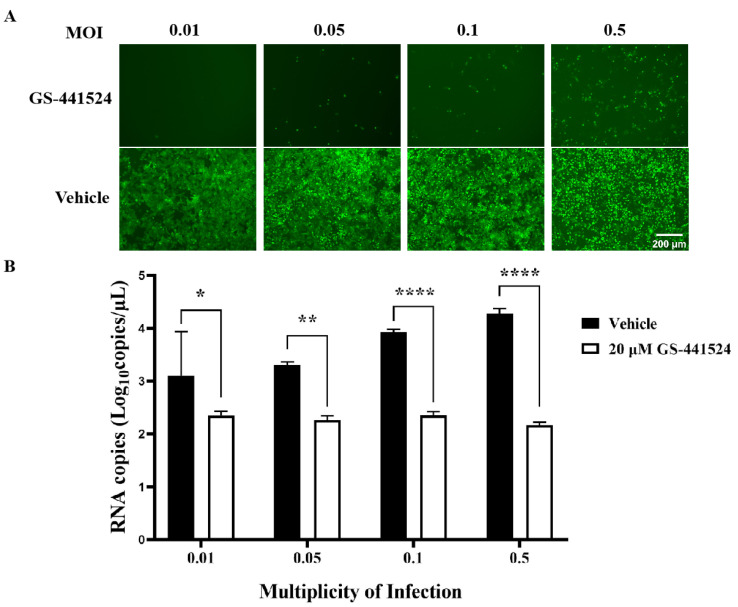
Antiviral effects of GS-441524 on PEDV under different infection doses. Vero cells were infected with rPEDV-∆ORF3-GFP of different MOIs and treated with 20 μM GS-441524 following the protocol designed to affect the PEDV replication phase. The GFP expression (**A**) and mRNA levels of M protein (**B**) were determined at 50 hpi. Data are presented as the mean ± SEM of three independent experiments. Differences were considered significant at (*) *p* < 0.05, (**) *p* < 0.01, (****) *p* < 0.0001. Scale bars = 200 μm.

In order to investigate the inhibitory effect of GS-441524 on PEDV infection at high infection doses, Vero cells were infected with rPEDV-∆ORF3-GFP at MOI 0.01, 0.05, 0.1, and 0.5, respectively, and treated with 20 μM GS-441524 following the protocol designed to affect the viral replication phase (Figure 5A). With the increase of viral infection dose from 0.01 to 0.5, the ratio of GFP-positive cells gradually increased, while GS-441524 significantly inhibited the ratio of GFP-positive cells, even at an MOI of 0.5 (Figure 6A). Compared with the control groups, where the mRNA levels of M protein increased with the increase of infection dose, the mRNA levels of M protein in the drug treatment groups remained almost unchanged and were significantly lower than those in the control groups (Figure 6B). In addition, viral titers could not be detected in the supernatant of any of the drug-treated groups (data not presented), indicating that even at high viral infection doses, 20 μM GS-441524 can effectively inhibit the proliferation of PEDV.

## 4. Discussion

GS-441524, like the active form of remdesivir in vivo, is phosphorylated into GS-441524 triphosphate by cell kinases, competing against the endogenous ATP for recognition and incorporation by the viral RNA-dependent RNA-polymerases (RdRp) [18]. To date, GS-441524 has been reported to exhibit broad-spectrum antiviral activity against a variety of RNA viruses, including a broad spectrum of coronaviruses, such as SARS-CoV [12], Middle Eastern respiratory syndrome coronavirus (MERS-CoV) [19], mouse hepatitis virus (MHV) [20], and other zoonotic coronaviruses [21]. GS-441524 treatment can rapidly reverse the disease symptoms of FIP, allowing sick cats to return to normal without significant toxicity [21]. GS-441524 and remdesivir potently inhibited SARS-CoV-2 replication in a dose-dependent manner, and GS-441524 had stronger anti-SARS-CoV-2 activity than remdesivir [22]. In addition, GS-441524 displayed a favorable oral bioavailability of 57% and produced adequate drug exposure in mouse plasma and lungs, suggesting that it could be a promising oral antiviral drug for the treatment of COVID-19 [13]. 

In this study, we investigated the antiviral activity of GS-441524 against trypsin-independent PEDV and confirmed that it could also significantly inhibit the proliferation of trypsin-independent PEDV, with an EC_50_ value of 2.6 μM and the highest SIs (selective indices) of 40.15. We further showed that GS-441524 was capable of markedly suppressing viral amplification and virion production of PEDV in a dose-dependent manner within the concentration of 1.25~20 μM, and 20 μM GS-441524 could almost completely inhibit the proliferation of PEDV. Given that in vitro proliferation of clinical strains of PEDV usually requires the addition of exogenous trypsin, we further investigated the inhibitory effect of GS-441524 on trypsin-dependent PEDV. In order to minimize the influence of genetic background, two PEDV strains, DR13^att^ strain (trypsin-independent PEDV) and rPEDV-S strain (trypsin-dependent PEDV), with different S genes (the determinant of trypsin-dependency) were selected. The results showed that GS-441524 could potently inhibit the proliferation of trypsin-dependent and trypsin-independent PEDVs tested, but had a stronger inhibitory effect on trypsin-independent PEDV strain. As the genetic backgrounds of the two PEDV strains, including the coding gene for RdRp, which is the target of GS-441524, are identical except for the S gene, we speculate that the differential inhibitory activities of GS-441524 on the trypsin-dependent and trypsin-independent PEDVs might be attributed to the influence of trypsin on drug transmembrane transport. The transmembrane transport of nucleoside analog drugs mainly relies on equilibrative nucleoside transporters (ENTs) [23]. Trypsin added during rPEDV-S cultivation may reduce the transport efficiency of GS-441524 by degrading ENTs or altering cell membrane structure, thereby reducing the drug’s inhibitory efficacy against trypsin-dependent PEDV. However, this hypothesis and its underlying mechanisms require further experimental verification.

It is well-established that GS-441524 inhibits the activity of RdRp in its triphosphate form. Consistent with this, we found that administering 20 μM GS-441524 six hours after PEDV entered Vero cells could completely inhibit virus proliferation, while administering it during the adsorption and entry stages of PEDV infection had no significant effect on virus titers and structural protein expression. In addition, 20 μM GS-441524 effectively inhibited the proliferation of rPEDV-∆ORF3-GFP with an MOI less than 0.5. Although the fluorescence signal of the GFP protein intensifies with an increase in viral infection dose, the virus titer could not be detected. Taken together, these results suggested that GS-441524 was a potential candidate drug for the treatment of PEDV infection.

Several studies have shown that nucleoside analogs hold the potential as antiviral drugs against PEDV. Specifically, molnupiravir, N6-methyladenosine, beta-D-N(4)-hydroxycytidine, and ribavirin have been shown to have a significant inhibitory effect on PEDV [10,24,25]. Xie’s research revealed that remdesivir nucleoside (RDV-N) was more efficacious (EC_50_, 0.31 μM) in Vero E6 cells than remdesivir (EC_50_, 0.74 μM) and beta-D-N(4)-hydroxycytidine (EC_50_, 1.17 μM) [15]. Huang et al. compared the anti-PEDV activity of molnupiravir and remdesivir and found that remdesivir has stronger anti-PEDV activity than molnupiravir [26]. In our study, GS-441524 showed strong anti-PEDV activity with an EC_50_ of 2.6 μM, which is higher than the EC_50_ value of 0.31 reported in a previous study [15]. The antiviral activity of the compound is closely related to the viral strain, cell line, and viral infection dose. In the two studies, the viral infection dose was the same, both being 0.01 MOI. However, the viral strains and cell lines used were different. This may lead to the difference in the EC_50_ values. In our study, GS-441524 showed a low cytotoxicity (CC_50_ = 104.4 μM), which was far lower than that of remdesivir (CC_50_, 1.7–15.0 μM) [27], indicating that GS-441524 has distinct advantages over remdesivir in treating PEDV infection. 

## 5. Conclusions

In summary, our study indicates that GS-441524 exhibits a favorable antiviral activity against trypsin-independent PEDV in vitro, with EC_50_ and CC_50_ values of 2.6 μM and 104.4 μM, respectively. It significantly inhibits the proliferation of both trypsin-dependent and trypsin-independent PEDV strains by suppressing the replication phase of the viral life cycle. Moreover, even when administered 6 h after the viruses enter the cells, 20 μM GS-441524 can completely inhibit the proliferation of PEDV. Although further research is needed to clarify the underlying mechanisms by which the trypsin-independent PEDV is more sensitive to GS-441524 than the trypsin-dependent strain, our findings suggest that GS-441524 is a potential drug for treating PEDV infection. These findings provide valuable new insight into the anti-PEDV activity of GS-441524 and enhance our understanding of nucleoside analogue drug-virus interactions. 

## Data Availability

The original contributions presented in this study are included in the article. Further inquiries can be directed to the corresponding authors.

## References

[1] Wang D., Fang L., Xiao S. (2016). Porcine epidemic diarrhea in China. Virus Res..

[2] Pensaert M.B., Bouck P.d. (1978). A new coronavirus-like particle associated with diarrhea in swine. Arch. Virol..

[3] Pensaert M.B., Martelli P. (2016). Porcine epidemic diarrhea: A retrospect from Europe and matters of debate. Virus Res..

[4] Vlasova A.N., Marthaler D., Wang Q., Culhane M.R., Rossow K.D., Rovira A., Collins J., Saif L.J. (2014). Distinct Characteristics and Complex Evolution of PEDV Strains, North America, May 2013–February 2014. Emerg. Infect. Dis.

[5] Jang G., Lee D., Shin S., Lim J., Won H., Eo Y., Kim C.H., Lee C. (2023). Porcine epidemic diarrhea virus: An update overview of virus epidemiology, vaccines, and control strategies in South Korea. J. Vet. Sci..

[6] Lei J., Miao Y., Bi W., Xiang C., Li W., Zhang R., Li Q., Yang Z. (2024). Porcine epidemic diarrhea virus: Etiology, epidemiology, antigenicity, and control strategies in China. Animals.

[7] Izes A.M., Yu J., Norris J.M., Govendir M. (2020). Current status on treatment options for feline infectious peritonitis and SARS-CoV-2 positive cats. Vet. Q..

[8] Brown A.J., Won J.J., Graham R.L., Dinnon K.H., Sims A.C., Feng J.Y., Cihlar T., Denison M.R., Baric R.S., Sheahan T.P. (2019). Broad spectrum antiviral remdesivir inhibits human endemic and zoonotic deltacoronaviruses with a highly divergent RNA dependent RNA polymerase. Antivir. Res..

[9] Sheahan T.P., Sims A.C., Graham R.L., Menachery V.D., Gralinski L.E., Case J.B., Leist S.R., Pyrc K., Feng J.Y., Trantcheva I. (2017). Broad-spectrum antiviral GS-5734 inhibits both epidemic and zoonotic coronaviruses. Sci. Transl. Med..

[10] Huang Z., Zhou S., Yang Z., Wang Z. (2023). Molnupiravir inhibits porcine epidemic diarrhea virus infection in vitro. Viruses.

[11] Krentz D., Zwicklbauer K., Felten S., Bergmann M., Dorsch R., Hofmann-Lehmann R., Meli M.L., Spiri A.M., Both U., Alberer M. (2022). Clinical follow-up and postmortem findings in a cat that was cured of feline infectious peritonitis with an oral antiviral drug containing GS-441524. Viruses.

[12] Pitts J., Li J., Perry J.K., Pont V.D., Riola N., Rodriguz L., Lu X., Kurhade C., Xie X., Camus G. (2022). Remdesivir and GS-441524 retain antiviral activity against Delta, Omicron, and other emergent SARS-CoV-2 variants. Antimicrob. Agents Chemother..

[13] Xie J., Wang Z. (2021). Can remdesivir and its parent nucleoside GS-441524 be potential oral drugs? An in vitro and in vivo DMPK assessment. Acta. Pharm. Sin. B.

[14] Zhu J., Li Y., Liang J., Mubareka S., Slutsky A.S., Zhang H. (2022). The poteintial protective role of GS-441524, a metabolite of the prodrug remdesivir, in vaccine breakthrough SARS-CoV-2 infections. Intensive Care Res..

[15] Xie Y., Guo X., Hu T., Wei D., Ma X., Wu J., Huang B., Shen J. (2021). Significant inhibition of porcine epidemic diarrhea virus in vitro by Remdesivir, its parent nucleoside and β-D-N4 -hydroxycytidine. Virol. Sin..

[16] Li C., Li Z., Zou Y., Wicht O., van Kuppeveld F.J., Rottier P.J., Bosch B.J. (2013). Manipulation of the porcine epidemic diarrhea virus genome using targeted RNA recombination. PLoS ONE.

[17] Reed L.J., Muench H. (1938). A simple method of estimating fifty per cent endpoints. Am. J. Epidemiol..

[18] Moorthy R., Kennelly S.A., Rodriguez D.J., Harki D.A. (2021). An efficient synthesis of RNA containing GS-441524: The nucleoside precursor of remdesivir. RSC Adv..

[19] Sheahan T.P., Sims A.C., Leist S.R., Schafer A., Won J., Brown A.J., Montgomery S.A., Hogg A., Babusis D., Clarke M.O. (2020). Comparative therapeutic efficacy of remdesivir and combination lopinavir, ritonavir, and interferon beta against MERS-CoV. Nat. Commun..

[20] Zhou Q., Luo Y., Zhu Y., Chen Q., Qiu J., Cong F., Li Y., Zhang X. (2023). Nonsteroidal anti-inflammatory drugs (NSAIDs) and nucleotide analog GS-441524 conjugates with potent in vivo efficacy against coronaviruses. Eur. J. Med. Chem..

[21] Murphy B.G., Perron M., Murakami E., Bauer K., Park Y., Eckstrand C., Liepnieks M., Pedersen N.C. (2018). The nucleoside analog GS-441524 strongly inhibits feline infectious peritonitis (FIP) virus in tissue culture and experimental cat infection studies. Vet. Microbiol..

[22] Li Y., Cao L., Li G., Cong F., Li Y., Sun J., Luo Y., Chen G., Li G., Wang P. (2022). Remdesivir Metabolite GS-441524 Effectively Inhibits SARS-CoV-2 Infection in Mouse Models. J. Med. Chem..

[23] Reyes G., Naydenova Z., Abdulla P., Chalsev M., Villani A., Rose J.B., Chaudary N., DeSouza L., Siu K.W.M., Core I.R. (2010). Characterization of mammalian equilibrative nucleoside transporters (ENTs) by mass spectrometry. Protein Expr. Purif..

[24] Chen J., Jin L., Wang Z., Wang L., Chen Q., Cui Y., Liu G. (2020). N6-methyladenosine regulates PEDV replication and host gene expression. Virology.

[25] Kim Y., Lee C. (2013). Ribavirin efficiently suppresses porcine nidovirus replication. Virus Res..

[26] Huang Z., Zhou S., Wang J., Yang Z., Wang Z. (2023). Remdesivir inhibits Porcine epidemic diarrhea virus infection in vitro. Heliyon.

[27] Warren T.K., Jordan R., Lo M.K., Ray A.S., Mackman R.L., Soloveva V., Siegel D., Perron M., Bannister R., Hui H.C. (2016). Therapeutic efficacy of the small molecule GS-5734 against Ebola virus in rhesus monkeys. Nature.

